# The Complete Plastome Sequences of Seven Species in *Gentiana* sect. *Kudoa* (Gentianaceae): Insights Into Plastid Gene Loss and Molecular Evolution

**DOI:** 10.3389/fpls.2018.00493

**Published:** 2018-05-01

**Authors:** Shan-Shan Sun, Peng-Cheng Fu, Xiao-Jun Zhou, Yan-Wei Cheng, Fa-Qi Zhang, Shi-Long Chen, Qing-Bo Gao

**Affiliations:** ^1^College of Life Science, Luoyang Normal University, Luoyang, China; ^2^Key Laboratory of Adaptation and Evolution of Plateau Biota, Northwest Institute of Plateau Biology, Chinese Academy of Sciences, Xining, China; ^3^Qinghai Provincial Key Laboratory of Crop Molecular Breeding, Xining, China

**Keywords:** evolution, gene loss, *Gentiana*, sect. *Kudoa*, plastome

## Abstract

The chloroplast (cp) genome is useful in the study of phylogenomics, molecular dating, and molecular evolution. *Gentiana* sect. *Kudoa* is a predominantly alpine flowering plant that is valued for its contributions to medicine, ecology, and horticulture. Previous evolutionary studies showed that the plastid gene loss pattern and intra-sectional phylogenetics in sect. *Kudoa* are still unclear. In this study, we compared 11 *Gentiana* plastomes, including 7 newly sequenced plastomes from sect. *Kudoa*, to represent its three serious: ser. *Ornatae*, ser. *Verticillatae*, and ser. *Monanthae*. The cp genome sizes of the seven species ranged from 137,278 to 147,156 bp. The plastome size variation mainly occurred in the small single-copy and long single-copy regions rather than the inverted repeat regions. Compared with sect. *Cruciata*, the plastomes in ser. *Ornatae* and ser. *Verticillatae* had lost approximately 11 kb of sequences containing 11 *ndh* genes. Conversely, far fewer losses were observed in ser. *Monanthae.* The phylogenetic tree revealed that sect. *Kudoa* was not monophyletic and that ser. *Monanthae* was more closely related to other sections rather than sect. *Kudoa.* The molecular dating analysis indicated that ser. *Monanthae* and sect. *Kudoa* diverged around 8.23 Ma. In ser. *Ornatae* and ser. *Verticillatae*, the divergence occurred at around 0.07–1.78 Ma. The nucleotide diversity analysis indicated that the intergenic regions *trnH-psbA*, *trnK-trnQ*, *ycf3-trnS* and *rpl32-trnL* constituted divergence hotspots in both sect. *Kudoa* and *Gentiana*, and would be useful for future phylogenetic and population genetic studies.

## Introduction

Encompassing 15 sections and 362 species (Ho and Liu, 2001), *Gentiana* is the largest genus in the family Gentianaceae (He, 1988). *Gentiana* is predominantly alpine and occurs in numerous mountain systems of the world. Hosting c. 250 species (Ho and Pringle, 1995), the mountain ranges surrounding the Qinghai-Tibetan Plateau (QTP) are the main diversity center of *Gentiana*. *Gentiana* has been widely used in traditional Chinese and Tibetan medicines and acts as an edificator in the QTP alpine meadows (Ho and Liu, 2001). In light of their chemical and horticultural value, several species have already been cultured (Rybczyński et al., 2015). Section *Kudoa* (Masamune) Satake & Toyokuni ex Toyokuni is characterized by roots arising from a collar and stems branching monopodially. This section contains three series and 28 species (Ho and Liu, 2001). Series *Ornatae* Marquand is the biggest series in sect. *Kudoa* and contains 16 species. The species in ser. *Ornatae* have showy flowers, and several of them have been domesticated for horticultural gardening (Rybczyński et al., 2015). ser. *Monanthae* (H. Smith) T. H. Ho and Series *Verticillatae* Marquand contain four and eight species, respectively (Ho and Liu, 2001).

A comparative analysis of the chloroplast genomes between sect. *Kudoa* and sect. *Cruciata* Gaudin indicated a loss of an approximately 10 kb sequence that mainly comprised 11 *ndh* genes in *Gentiana lawrencei* var. *farreri*, which belongs to ser. *Ornatae* (Fu et al., 2016). Variable *ndh* gene loss has been reported in other plant groups such as orchid (Barrett and Davis, 2012; Yang et al., 2013; Lin et al., 2015; Niu et al., 2017). To further assess whether the *ndh* gene has been lost in sect. *Kudoa*, additional taxa should be sequenced and included in the comparative analysis. In addition, the phylogenetic relationships in sect. *Kudoa* are currently controversial. In the latest classification system proposed by Ho and Liu (2001), sect. *Kudoa* contained three series. A phylogenetic study of the subtribe Gentianinae based on the intergenic spacer (ITS) region and a plastid fragment included only four taxa from sect. *Kudoa*, which clustered into a monophyletic group (Favre et al., 2010). In another phylogenetic study on *Gentiana* based on ITS and two plastid fragments, 18 taxa from sect. *Kudoa* were included and grouped into three clades (Favre et al., 2016), indicating that sect. *Kudoa* was not monophyletic, and that ser. *Verticillatae* was embedded in ser. *Ornatae*. The molecular phylogenetic relationship is not consistent with the classification system proposed by Ho and Liu (2001). Whether the plastid *ndh* gene loss pattern in sect. *Kudoa* is helpful to the intra-sectional phylogenetics is worth exploring.

The cp genomes are circular DNA molecules in angiosperms that range in size from 120 to 160 kb and contain 110–130 genes (Palmer, 1985). In land plants, the cp genome typically contains a pair of inverted repeats (IRs) that separate the remaining regions into one large single-copy region (LSC) and one small single-copy region (SSC) (Palmer, 1985; Jansen et al., 2005). The cp genome is recognized as the “workhorse” in plant systematics research due to its uniparental inheritance, haploid nature, highly conserved structures, and slower evolutionary rate of change compared to nuclear genomes (Wolfe et al., 1987; Shaw et al., 2014). Plastid phylogenomics has been widely applied to reassess classifications, for example, the reassessment of Alismatales (Ross et al., 2015), Rosaceae (Zhang S.D. et al., 2017), *Gaultheria* series *Trichophyllae* (Zhang M.Y. et al., 2017), and *Leptaspis* and *Streptochaeta* in Poaceae (Burke et al., 2016). In addition to phylogenetic classification, the cp genome is widely used in studies of molecular identification, divergence dating, and molecular evolution (Nikiforova et al., 2013; Carbonell-Caballero et al., 2015). Comparative cp genome analysis can reveal insights into the evolution of the cp genome, including sequence inversion (Cho et al., 2015) and gene loss (Wakasugi et al., 1994; Millen et al., 2001), and has been used in the identification of mutational hotspots for the screening of the most informative regions (e.g., Ahmed et al., 2013; Niu et al., 2017).

Presently, only four complete cp genomes have been sequenced in *Gentiana*, in which three belong to sect. *Cruciata* (Ni et al., 2016a,b) and one belongs to sect. *Kudoa* (Fu et al., 2016). The development of more genomic resources for *Gentiana* should inform our understanding of the phylogenetic relationships and evolutionary history of this large genus. In this study, we focused on sect. *Kudoa* and sequenced the complete cp genomes of seven species in this section. Based on a comparative analysis of these seven species, as well as four species in sect. *Cruciata* and sect. *Kudoa* with available genomes, the genome structure, gene loss, phylogenetic relationships, divergence times and mutational hotspots of sect. *Kudoa* were analyzed to discover (1) gene loss pattern, particularly *ndh*, in sect. *Kudoa*, and (2) plastome phylogenetic implication in sect. *Kudoa*. This study also makes available sequence information for phylogenetic and evolutionary studies of *Gentiana*.

## Materials and Methods

### Sample Collection, Genome Sequencing, and Assembly

A total of seven species were sampled in the QTP (Supplementary Table S1) to represent all three series of sect. *Kudoa*. Five species (*G. veitchiorum* Hemsley, *G. ornata* Grisebach, *G. caelestis* H. Smith, *G. obconica* T. N. Ho, and *G. oreodoxa* H. Smith) belong to ser. *Ornatae*, one (*G. stipitata* Edgeworth) belongs to ser. *Monanthae*, and one (*G. hexaphylla* Maximowicz ex Kusnezow) belongs to ser. *Verticillatae*. The species were identified by Dr. Peng-Cheng Fu and Dr. Shi-Long Chen. Voucher specimens were deposited in the herbarium of the College of Life Science, Luoyang Normal University. The samples were collected from a single plant of each species. Total genomic DNA isolation, DNA fragmentation, and sequencing library construction followed the process described in Fu et al. (2016). Based on the genome size of some *Gentiana* taxa (Mishiba et al., 2009) and reported examples of sequenced cp genomes (Fu et al., 2016), we expected to obtain approximately 5 Gb raw data for each species. The fragmented genomic DNA of the seven *Gentiana* species was sequenced using the Illumina HiSeq 4000 platform (Novogene, Tianjing, China), yielding 150-bp paired-end reads from a library of approximately 300-bp DNA fragments.

Reads corresponding to plastid DNA were identified using a BLASTN (*E*-value: 10^-6^) search against the plastome sequences of *G. lawrencei* var. *farreri* (GenBank accession no. KX096882). The recovered reads were assembled using Velvet 1.2.10 (Zerbino and Birney, 2008). Detailed information regarding the raw reads for each taxon is presented in Supplementary Table S2. All the genomic regions located at the junction between the two assembled contigs were verified by Sanger sequencing. The primers used were designed by PRIMER V. 5.0 software and are listed in Supplementary Table S3. The plastome sequences of the seven species were deposited in GenBank (MG192304–MG192310).

### Genome Annotation

For each species, the protein coding genes (PCGs), rRNAs, and tRNAs in the cp genome were predicted and annotated using Dual Organellar GenoMe Annotator (DOGMA) using the default parameters (Wyman et al., 2004). The positions of the start and stop codons, or intron/exon junctions of the PCGs, were manually corrected using a BLAST search against reported cp genomes of other closely related species. The cp gene maps of the seven species were drawn using OGDraw V. 1.2 (Lohse et al., 2007).

### Comparative Analysis

In addition to the seven newly sequenced species, the cp genome sequences of *G. lawrencei* var. *farreri* (KX096882), which belongs to ser. *Ornata*, and *G. straminea* (KJ657732), *G. robusta* (KT159969), and *G. crassicaulis* (KJ676538), which belong to sect. *Cruciata*, were obtained for comparative analysis from the National Center for Biotechnology Information. Genome comparisons were performed to identify the differences among the 11 taxa using mVISTA (Frazer et al., 2004) and Geneious Basic 5.6.4 (Kearse et al., 2012). To identify divergence hotspots, nucleotide diversity (Pi) was determined using DnaSP V. 5.10 (Librado and Rozas, 2009).

### Phylogenetic Analysis

To investigate the phylogenetic relationships of the genome sequences of sect. *Kudoa*, an additional 14 available complete cp genomes in the order Gentianales were retrieved from GenBank (Supplementary Table S4). Forty-six PCGs (*atp*A, *atp*B, *atp*E, *atp*H, *atp*I, *cem*A, *mat*K, *pet*A, *pet*B, *pet*D, *pet*G, *pet*L, *pet*N, *psa*A, *psa*B, *psa*I, *psa*J, *psb*A, *psb*C, *psb*D, *psb*E, *psb*F, *psb*H, *psb*I, *psb*I, *psb*K, *psb*L, *psb*M, *psb*N, *psb*T, *rbc*L, *rpl*14, *rpl*16, *rpl*20, *rpl*22, *rpl*33, *rpl*36, *rpo*A, *rps*2, *rps*3, *rps*4, *rps*8, *rps*11, *rps*14, *rps*15, and *rps*18) present in all of the species were extracted from the selected cp genomes. Phylogenetic analyses were performed using the concatenated nucleotide sequences and PhyML3.1 software (Guindon and Gascuel, 2003) using the maximum likelihood (ML) method. Based upon the Akaike information criterion in the software jModelTest 2.1.7 (Guindon and Gascuel, 2003; Posada, 2008), the selected best-fitting model of sequence evolution was the GTR+I+G model with a p-inv of 0.404 and gamma shape of 0.808. A bootstrap analysis was performed with 100 replications.

### Molecular Dating

The PCG dataset was used to estimate divergence times using the Bayesian method implemented in the program BEAST 1.7.5 (Drummond et al., 2012) under the GTR+I+G substitution model, the Yule model, and an uncorrelated lognormal clock model (Drummond et al., 2006). Due to the limited fossils available for *Gentiana*, we constrained only one of the nodes with a seed fossil of sect. *Cruciata.* For the seed fossil, we used lognormal priors with an offset at 5.0 Ma, a mean of 0.7, and a standard deviation of 1.0, as applied by Pirie et al. (2015) and Favre et al. (2016). We did not use uniform priors for sect. *Cruciata,* as they were rejected following comparison with the lognormal priors provided by Favre et al. (2016). We ran three independent Markov chain Monte Carlo analyses for 50 million generations, sampling every 5,000th generation. We assessed the convergence of the estimated parameters in Tracer 1.5 (Rambaut and Drummond, 2010), ensuring that the effective sample size values exceeded 200. Trees were summarized in TreeAnnotator 1.7.5 (Drummond et al., 2012) after setting the burn-in to 10%, and then visualized in FigTree 1.4.0^1^.

## Results

### Features of the Seven Newly Sequenced Plastomes

Complete plastome sequences of seven *Gentiana* species were newly sequenced in this study and deposited in GenBank. The seven plastid genomes constituted closed circular molecules whose sizes ranged from 137,278 to 147,156 bp with an average of 138,822 bp (**Table 1**). Each cp genome comprised a pair of IR regions (IRa and IRb), one LSC region, and one SSC region. They all possessed the overall typical quadripartite structure that resembles the majority of land plant cp genomes (Shinozaki et al., 1986). The IR regions of the 7 species ranged from 23,864 to 25,229 bp; the LSC regions ranged from 77,754 to 79,712 bp; and the SSC regions ranged from 11,353 to 16,986 bp (**Table 1**). *Gentiana stipitata* possessed the longest LSC, SSC, and IR regions of the seven species. The average GC contents of the LSC, SSC, and IR regions and the whole cp genome in the seven species were 35.7, 30.6, 43.8, and 36.8%, respectively, which corroborates other reported *Gentiana* cp genomes (Fu et al., 2016; Ni et al., 2016a,b). Furthermore, these plastid genomes were similar in structure and gene arrangement to previously published *Gentiana* plastomes (Fu et al., 2016; Ni et al., 2016a,b). All the plastome maps are presented in Supplementary Figures S1–S7.

**Table 1:** Base composition of the chloroplast genomes in *Gentiana*.

Species	Taxonomic treatment	GenBank number	LSC (bp)	IR (bp)	SSC (bp)	Total (bp)
*G. caelestis*	sect. *Kudoa* ser. *Ornatae*	MG192304	77,870	24,113	11,548	137,644
*G. obconica*	sect. *Kudoa* ser. *Ornatae*	MG192306	77,754	23,865	11,794	137,278
*G. oreodoxa*	sect. *Kudoa* ser. *Ornatae*	MG192307	77,908	23,865	11,765	137,403
*G. ornata*	sect. *Kudoa* ser. *Ornatae*	MG192308	77,816	24,108	11,353	137,385
*G. veitchiorum*	sect. *Kudoa* ser. *Ornatae*	MG192310	77,932	23,864	11,807	137,467
*G. lawrencei* var. *farreri*	sect. *Kudoa* ser. *Ornatae*	KX096882	78,082	24,635	11,365	138,750
*G. hexaphylla*	sect. *Kudoa* ser. *Verticillatae*	MG192305	77,922	23,865	11,771	137,423
*G. stipitata*	sect. *Kudoa* ser. *Monanthae*	MG192309	79,712	25,229	16,986	147,156
*G. crassicaulis*	sect. *Cruciata*	KJ676538	81,164	25,271	17,070	148,776
*G. robusta*	sect. *Cruciata*	KT159969	81,164	25,333	17,081	148,991
*G. straminea*	sect. *Cruciata*	KJ657732	81,240	25,333	17,085	148,991


*The chloroplast genomes of sect. Cruciata were downloaded from GenBank, while the remainder were sequenced in this study.*

### Comparison of cp Genomes

**FIGURE 1:**
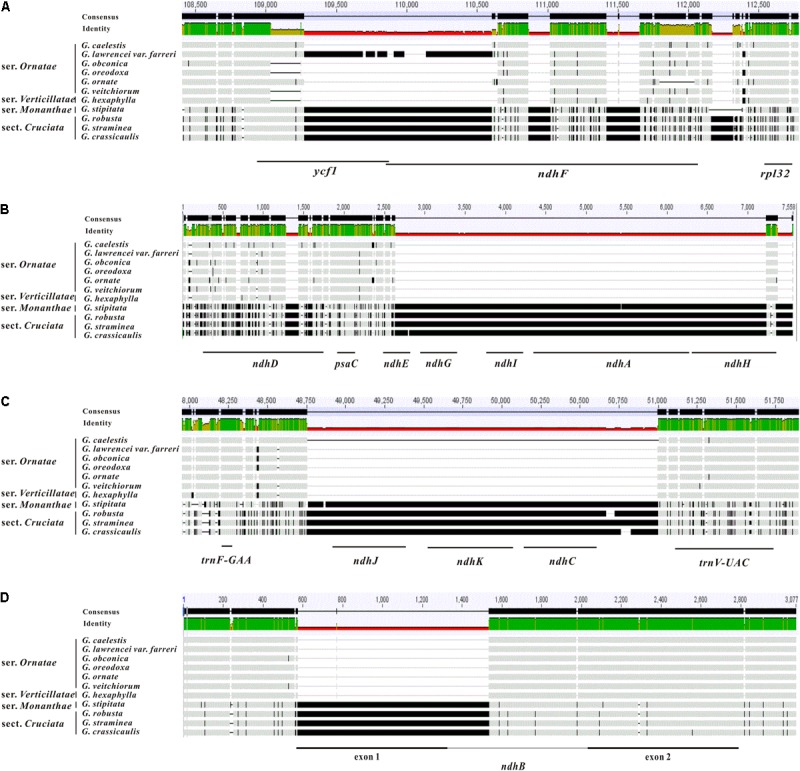
The sequence length variation of four regions across 11 *Gentiana* chloroplast genomes. **(A)** The region between *ycf1* and *rpl32*; **(B)** the region between *ccs*A and *rps*15; **(C)** the region between *trn*F-GAA and *trn*V-UAC; **(D)** the region between *trn*L*-*CAA and *rps*7.

The comparative analysis indicated that the six species in *Gentiana* ser. *Ornatae* possessed very similar plastomes, with genome sizes ranging from 137,278 to 138,750 bp. The only obvious difference was located at the boundary between IRb and SSC (**Figure 1A**), in which four sequence patterns were detected. The first pattern, which appeared in *G.* *lawrencei* var. *farreri*, possessed almost all the sequences of *ycf*1 and most of the forward sequences of *ndh*F (**Figure 1A**). The second pattern, appearing in *G. caelestis* and *G. ornata*, possessed the forward 416 bp of *ycf*1, but had lost the forward sequence of *ndh*F (**Figure 1A**). In comparison with the second pattern, the third pattern only possessed the forward 181 bp of *ycf*1 (**Figure 1A**). The third pattern appeared in *G. obconica*, *G. veitchiorum,* and *G. oreodoxa*. In sect. *Kudoa*, *G. hexaphylla* from ser. *Verticillatae* also possessed the third pattern. However, *G. stipitata* from ser. *Monanthae* possessed all of the whole sequences of *ycf*1 and *ndh*F, constituting the fourth pattern. This species differed from all the other taxa in sect. *Kudoa*, but did not differ from the three taxa in sect. *Cruciata* (**Figure 1A**).

The comparative analysis in *Gentiana* revealed that the variation in plastid genome size in the 11 plastomes could mainly be attributed to sequence loss in four locations. One of these locations was the IRb-SSC boundary mentioned above. The second was the region between *ccs*A to *rps*15, in which about 5 kb had been lost in some taxa. The lost sequences mainly contained a small section of *ndh*D, the majority of *ndh*E and *ndh*H, and all of *ndh*G, *ndh*I, and *ndh*A (**Figure 1B**). These *ndh* genes are shown in yellow in the Supplementary Figures S1–S7. The third was the region between *trn*F-GAA and *trn*V-UAC, in which about 2.2 kb had been lost in some taxa. The lost sequences mainly contained all of *ndh*J, *ndh*K, and *ndh*C (**Figure 1C**). The fourth was the region between *trn*L*-*CAA and *rps*7, in which about 1 kb had been lost in some taxa. The lost sequences mainly contained the whole exon 1 and part of the intron of *ndh*B (**Figure 1D**). All the sequences that have been lost in the three regions were missing in the species of ser. *Ornatae* and ser. *Verticillatae,* but present in ser. *Monanthae* and sect. *Cruciata*.

### Phylogenetic and Molecular Dating Analyses

**FIGURE 2:**
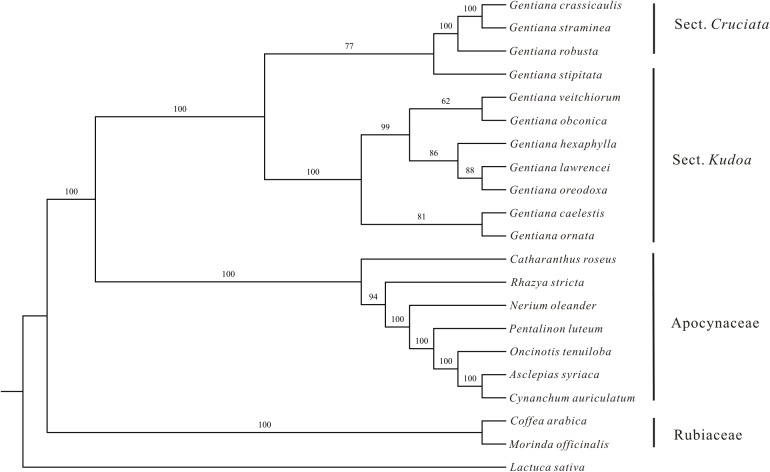
Maximum likelihood tree of Gentianales based on 46 PCGs of the chloroplast genomes. Numbers above the nodes indicate bootstrap support values.

The ML phylogenetic tree constructed using the 46 PCGs clearly identified the three families (Gentianaceae, Apocynaceae, and Rubiaceae) as being monophyletic with high bootstrap support (**Figure 2**). Two monophyletic groups were identified within the Gentianaceae clade in these analyses. Taxa from ser. *Ornatae* and ser. *Verticillatae* clustered into one monophyletic group whose monophyletic sister group contained taxa from sect. *Cruciata* and ser. *Monanthae* (**Figure 2**). The ML tree showed that *G. stipitata* was more closely related to other sections rather than sect. *Kudoa.*

**FIGURE 3:**
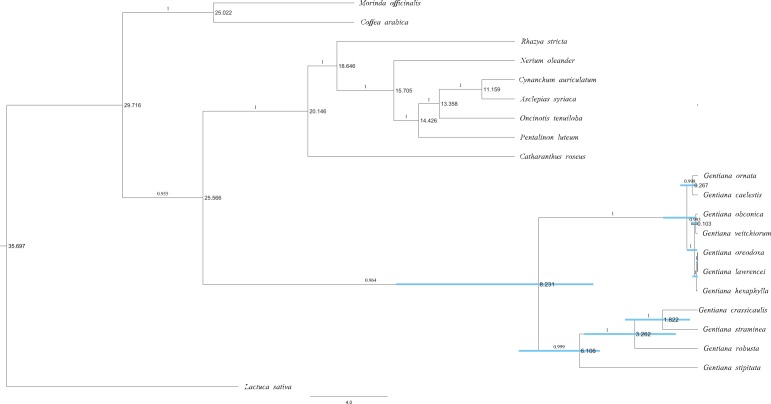
Majority-rule consensus phylogenetic tree of Gentianales based on 46 PCGs of the chloroplast genomes based on Bayesian inference. Numbers on the branches indicate Bayesian posterior probabilities. Node age estimates are marked dextrally. Blue bars represent 95% HPDs.

The molecular dating analysis of the PCG dataset (**Figure 3**) estimated that the two lineages in the *Gentiana* tree diverged approximately 8.23 Ma (95% highest posterior density [HPD]: 5.40–15.60 Ma). *G. stipitata* and sect. *Cruciata* diverged about 6.11 Ma (95% HPD: 5.06–9.25 Ma). The divergence in ser. *Ornatae* and ser. *Verticillatae* occurred at around 0.07–1.78 Ma.

### Divergence Hotspots in *Gentiana*

The coding genes, introns, and non-coding regions were compared to detect divergence hotspots. We compared all 11 species mentioned above as well as the 8 species in sect. *Kudoa*. A total of 114 regions (49 coding genes, 9 intron regions, and 55 intergenic regions) greater than 200 bp were generated in both comparisons.

**FIGURE 4:**
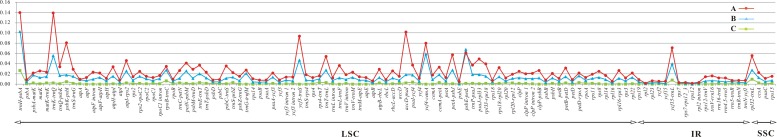
Comparison of the nucleotide variability (Pi) values in **(A)** *Gentiana*, **(B)** sect. *Kudoa*, and **(C)** sect. *Kudoa* except ser. *Monanthae*. A total of 114 regions (49 coding genes, 9 intron regions, and 55 intergenic regions) greater than 200 bp in size were generated in both comparisons.

Among the 11 species in *Gentiana*, Pi ranged from 0.00163 (*rpl2* gene) to 0.13984 (*trnH-psbA* region). The average Pi in LSC, SSC, and IR was 0.02696, 0.02624, and 0.011, respectively. Five regions (*trnH-psbA*, *trnK-trnQ*, *accD-psaI*, *ycf3-trnS,* and *psbK-psbI*), all located in the LSC region, showed high levels of variation (**Figure 4A** and **Table 2**).

**Table 2:** Five regions of highly variable sequences of *Gentiana*, sect. *Kudoa* and sect. *Kudoa* except ser. *Monanthae*.

	Nucleotide diversity (Pi)	Total number of mutation (Eta)
***Gentiana***		
*trnH-psbA*	0.13984	92
*trnK-trnQ*	0.13947	168
*accD-psaI*	0.10200	62
*ycf3-trnS*	0.09391	59
*psbK-psbI*	0.08074	54
**sect. *Kudoa***		
*trnH-psbA*	0.10232	79
*psbE-petL*	0.06795	172
*ycf4-cemA*	0.05877	71
*trnK-trnQ*	0.05557	157
*ycf3-trnS*	0.04688	57
**sect. *Kudoa* except ser. *Monanthae***		
*trnH-psbA*	0.02693	25
*rpl32-trnL*	0.00991	22
*ycf15-trnL*	0.00773	5
*rpoB-trnC*	0.00647	21
*psbK-psbI*	0.00503	3


The sequence divergence among the eight species in sect. *Kudoa* ranged from 0.00100 (*psbE* gene) to 0.10232 (*trnH-psbA* region). The average Pi in LSC, SSC, and IR was 0.01406, 0.01320, and 0.00543, respectively. Five of these, *trnH-psbA*, *psbE-petL*, *ycf4-cemA*, *trnK-trnQ*, and *ycf3-trnS*, constituted highly divergent hotspots (**Table 2**). All of the five regions were located in the LSC region (**Figure 4B**).

The sequence divergence among the seven species in sect. *Kudoa* except ser. *Monanthae* is much lower than the sequence divergence among sect. *Kudoa* and *Gentiana* (**Figure 4C**). The average Pi in LSC, SSC, and IR was 0.00145, 0.00362, and 0.00067, respectively. Five of these, *trnH-psbA*, *rpl32-trnL*, *ycf15-trnL*, *rpoB-trnC*, and *psbK-psbI*, constituted highly divergent hotspots (**Table 2**).

## Discussion

### Loss of Plastid *ndh* Genes in *Gentiana* sect. *Kudoa*

Variations in plastome length were detected in *Gentiana*. Compared with sect. *Cruciata*, ser. *Ornatae* and ser. *Verticillatae* lost approximately 11 kb of sequences, while ser. *Monanthae* lost almost none. The plastomes of ser. *Ornatae* and ser. *Verticillatae* were highly similar in size and structure. The majority of length variation of the newly sequenced plastomes of ser. *Ornatae* and ser. *Verticillatae* in this study mainly occurred in the SSC and LSC regions, rather than the two IR regions. The length variation pattern is similar to that observed in *G. lawrencei* var. *farreri* (Fu et al., 2016), whose genome size variation was not caused by deletions in the IR regions, but by deletions in the SSC and LSC regions. An explanation for the size variation pattern is that the junction between the IR and LSC region is located within the *rps19* gene, which is a coding gene, and thus contributes to the more constant size of the IRs than the SSC and LSC region in the great majority of angiosperms (Palmer, 1985; Fu et al., 2016). As noted in *G. lawrencei* var. *farreri* (Fu et al., 2016), the genome size variation led to the loss of plastid *ndh* genes.

Plastid *ndh* genes have been retained in the majority of higher plants (Martín and Sabater, 2010), and appear to have been frequently lost in parasitic and epiphytic plants (e.g., Stefanović and Olmstead, 2005). Along with the publication of numerous plastomes, the independent loss of *ndh* genes has been detected in increasing numbers of higher plants including orchids (Chang et al., 2006; Yang et al., 2013; Lin et al., 2015; Niu et al., 2017), gnetophytes (Braukmann et al., 2009; Wu et al., 2011), slender naiads (Peredo et al., 2013), and saguaros (Sanderson et al., 2015). The independent *ndh* gene loss in various groups could be an example of convergent evolution in plants. In *Gentiana*, the loss of *ndh* genes was previously detected in *G. lawrencei* var. *farreri*, which belongs to sect. *Kudoa*, but was not detected in the other three previously sequenced plastomes (Fu et al., 2016; Ni et al., 2016a). Upon analysis of the plastomes of sect. *Kudoa,* we discovered that the loss of *ndh* genes was common in ser. *Ornatae* and ser. *Verticillatae*. The 11 *ndh* genes in the plastome encode a protein complex that catalyzes the transfer of electrons from NADH to plastoquinone at photosystem I (Sazanov et al., 1998; Martín and Sabater, 2010). However, the PGR5-dependent cyclic electron transport pathway already exists in cells. Transgenic plants defective in *ndh* genes showed impaired photosynthesis rates, demonstrating that the NDH complex is required for the optimization of photophosphorylation rates and might play an important role in regulating CO_2_ assimilation under stress conditions (Wang et al., 2006; Martín and Sabater, 2010). However, no deleterious effects have been observed in *ndh*-deficient mutants under favorable growing conditions (Ruhlman et al., 2015). This suggests that the plastid *ndh* genes might be dispensable in contemporary plants. A plastome study in orchids proposed that the expansion/contraction of IR boundaries might be associated with the loss of *ndh* genes, especially *ndhF* (Kim et al., 2015; Niu et al., 2017). Previous studies have shown that the expansion of IRs is common to *ndh*-absent plastomes (Niu et al., 2017). In *Gentiana*, it is likely that the expansion/contraction of IR boundaries is correlated with the deletion of the *ndh* gene, particularly *ndhF*. However, the contraction, rather than expansion, of IRs was observed in *ndh*-deleted *Gentiana* plastomes. This suggests that the evolution of *ndh* genes in plastomes may vary between different taxa, and thus requires further exploration.

The loss of plastid *ndh* genes was common in sect. *Kudoa,* with the exception of ser. *Monanthae*. Compared with sect. *Cruciata*, ser. *Monanthae* did not exhibit significant size variation. As observed in sect. *Cruciata*, *G. stipitata* in ser. *Monanthae* maintained all 11 plastid *ndh* genes. This suggests that ser. *Monanthae* might be evolutionarily more closely related to other sections rather than sect. *Kudoa* (although cp genome sequences from additional sections are necessary to confirm this). Considering that gene loss in plastomes is an ongoing process in evolution (Martin et al., 1998), ser. *Ornatae* and ser. *Verticillatae* may have shorter evolutionary histories than ser. *Monanthae*.

### Phylogenetic Relationships and Divergence Times

Plastid phylogenomics has been successfully applied in the phylogenetic reassessment of several groups (Burke et al., 2016; Zhang M.Y. et al., 2017; Zhang S.D. et al., 2017). In this study, the phylogenetic relationships constructed using the 46 PCGs were consistent with previous studies whereby the three families (namely Gentianaceae, Apocynaceae, and Rubiaceae) were classified as three monophyletic clades, and also identified Rubiaceae as the base group in Gentianales (Backlund et al., 2000).

The delimitation of *Gentiana* sect. *Kudoa* has been controversial. Ho (1985) and Ho and Pringle (1995) erected four serious in sect. *Kudoa*: ser. *Monanthae*, ser. *Ornatae*, ser. *Verticillatae,* and ser. *Apteroideae* (H. Smith) T. N. Ho. Based on 61 informative characters from morphology, palynology, and cytology, Ho et al. (1996) supported three series in sect. *Kudoa* and treated ser. *Apteroideae* as sect. *Monopodiae* (H. Smith) T. N. Ho. The treatment of three series in sect. *Kudoa* was accepted by Ho and Liu (2001), and was also corroborated by the phylogenetic analysis of Favre et al. (2016) based on *atpB-rbcL*, *trnL–trnF,* and ITS. The taxa from sect. *Monopodiae*, namely *Kudoa* I in Favre et al. (2016), constituted a monophyletic group and were paraphyletic with the other sections (Favre et al., 2016). We therefore adopted the treatment of three series in sect. *Kudoa* as the starting point of this study.

Contrary to the classical classification, molecular phylogeny shows that sect. *Kudoa* is paraphyletic. Although the phylogeny of sect. *Kudoa* was not mainly discussed in Favre et al. (2016), the phylogenetic trees in their study also indicated that the three series are not a monophyletic group. Our plastid phylogenomic relationships is in accord with this. The cp-based phylogenomic tree suggested that ser. *Monanthae* was more closely related to other sections rather than sect. *Kudoa*. The previous phylogenetic trees reconstructed based on *atpB-rbcL*, *trnL–trnF* and ITS data indicated that ser. *Monanthae*, sect. *Isomeria* and sect. *Microsperma* clustered into one clade (Favre et al., 2016). The instability of ser. *Monanthae* in the phylogenetic tree may suggest a potential hybrid origin of this group, since they have a partly sympatric distribution and nearly contemporaneous flowering times to other groups like sect. *Kudoa* and sect. *Cruciata* (Ho and Liu, 2001), and furthermore, hybridization is an important means of speciation in the QTP (Li et al., 2007; Liu et al., 2014; Wen et al., 2014). In terms of morphology, ser. *Verticillatae* is characterized by whorled phyllotaxy. However, our phylogenomic tree showed that ser. *Verticillatae* did not constitute a monophyletic group, but was rather inlaid in ser. *Ornatae*. This is also supported by the phylogenetic tree based on *atpB-rbcL*, *trnL–trnF,* and ITS (Favre et al., 2016). The results suggested that the present classification in sect. *Kudoa* based on morphology is not consistent with the molecular phylogenetic reconstruction. Therefore, the phylogenetic relationships in sect. *Kudoa* require further evaluation.

Molecular dating analysis in this study showed that ser. *Monanthae* and sect. *Cruciata* diverged around 6.11 Ma (95% HPD: 5.06–9.25 Ma) when sect. *Cruciata* diverged from its sister groups (Favre et al., 2016). The divergence of the ser. *Verticillatae* and ser. *Ornatae* clade occurred at around 0.07–1.78 Ma, which is much younger than 1.34–9.55 Ma estimated in Favre et al. (2016). Since the *ndh* gene loss indicated that ser. *Verticillatae* and ser. *Ornatae* were younger than ser. *Monanthae*, we believe that the time of divergence between ser. *Verticillatae* and ser. *Ornatae* found in this study may be true. We recommend that the plastomes and *ndh* gene remain/loss patterns of more taxa that are closely related to sect. *Kudoa* are included in future studies.

### Mutational Hotspot

The comparative analysis indicated that the plastomes of ser. *Ornatae* and ser. *Verticillatae* exhibited little variation. The popular barcoding plastid markers such as *matK* and *rbc*L (Li et al., 2015; Coissac et al., 2016) exhibited very poor sequence variation in the two series. Some popular genetic markers used in intra-species population genetics such as *trnS-trnG*, *rps15-ycf1,* and *rpl20-rps12* (e.g., Burnier et al., 2009; Takayama et al., 2013; Khan et al., 2016) show very limited variation in the populations of some species such as *G. veitchiorum* and *G. lawrencei* var. *farreri* (Fu, Unpublished Data). Additionally, *trnQ-rps16* (Shepherd et al., 2017) was not detected as *rps16* is a pseudogene in *Gentiana* (Ni et al., 2016a). Since the plastome-wide comparisons could facilitate the screening of mutational hotspots used for inter-species phylogenetics (Shaw et al., 2014) and intra-species discrimination (Ahmed et al., 2013), suitable molecular markers for phylogenetic and population genetic studies could be identified in the mutational hotspots in *Gentiana*, particularly in sect. *Kudoa.*

The identified sequence divergence hotspot regions in *Gentiana* and sect. *Kudoa* in this study were *trnH-psbA*, *trnK-trnQ*, *accD-psaI*, *ycf3-trnS*, *psbK-psbI*, *psbE-petL,* and *ycf4-cemA.* Focusing on ser. *Ornatae* and ser. *Verticillatae*, the divergence hotspot regions were *trnH-psbA*, *rpl32-trnL*, *ycf15-trnL*, *rpoB-trnC*, and *psbK-psbI*. These findings should inform future studies on the inter-species phylogenetics and intra-species population genetics in *Gentiana* and sect. *Kudoa*.

## Conclusion

The complete cp genome sequences of seven species from *Gentiana* sect. *Kudoa* were reported in this study, and the evolutionary characteristics of 11 *Gentiana* plastomes from two sections were described. We discovered that the loss of plastid *ndh* genes is common in ser. *Ornatae* and ser. *Verticillatae,* but not in ser. *Monanthae.* The phylogenetic tree and deletion patterns in the plastid *ndh* genes indicated that ser. *Monanthae* is more closely related to other sections rather than sect. *Kudoa,* which is not monophyletic. The sequence and divergence hotspot information presented here could be useful in future studies on the population genetics, phylogenetics, and evolution of *Gentiana*.

## Author Contributions

S-SS designed the experiments, performed the experiments, analyzed the data, prepared the figures and/or tables, and revised the manuscript. P-CF conceived and designed the experiments, performed the experiments, analyzed the data, wrote the paper, and prepared the figures and/or tables. X-JZ and Y-WC performed the experiments and analyzed the data. F-QZ and S-LC collected the samples and reviewed the drafts of the paper. Q-BG analyzed the data, prepared figures and/or tables, reviewed the drafts of the paper, and revised the manuscript.

## Conflict of Interest Statement

The authors declare that the research was conducted in the absence of any commercial or financial relationships that could be construed as a potential conflict of interest.

## Footnotes

1 http://tree.bio.ed.ac.uk/software/figtree/

## References

[B1] Ahmed I. Matthews P. J. Biggs P. J. Naeem M. Mclenachan P. A. Lockhart P. J. (2013). Identification of chloroplast genome loci suitable for high-resolution phylogeographic studies of *Colocasia esculenta* (L.) Schott (Araceae) and closely related taxa. *Mol. Ecol. Resour.* 13 929–937. 10.1111/1755-0998.12128 23718317

[B2] Backlund M. Oxelman B. Bremer B. (2000). Phylogenetic relationships within the Gentianales based on ndhF and rbcL sequences, with particular reference to the Loganiaceae. *Am. J. Bot.* 87 1029–1043. 10.2307/2657003 10898781

[B3] Barrett C. F. Davis J. I. (2012). The plastid genome of the mycoheterotrophic *Corallorhiza striata* (Orchidaceae) is in the relatively early stages of degradation. *Am. J. Bot.* 99 1513–1523. 10.3732/ajb.1200256 22935364

[B4] Braukmann T. W. Kuzmina M. Stefanović S. (2009). Loss of all plastid ndh genes in Gnetales and conifers: extent and evolutionary significance for the seed plant phylogeny. *Curr. Genet.* 55 323–337. 10.1007/s00294-009-0249-7 19449185

[B5] Burke S. V. Lin C. S. Wysocki W. P. Clark L. G. Duvall M. R. (2016). Phylogenomics and plastome evolution of tropical forest grasses (*Leptaspis, Streptochaeta*: Poaceae). *Front. Plant. Sci.* 7:1993. 10.3389/fpls.2016.01993 28083012 PMC5186769

[B6] Burnier J. Buerki S. Arrigo N. Küpfer P. Alvarez N. (2009). Genetic structure and evolution of Alpine polyploid complexes: *Ranunculus kuepferi* (Ranunculaceae) as a case study. *Mol. Ecol.* 18 3730–3744. 10.1111/j.1365-294X.2009.04281.x 19674303

[B7] Carbonell-Caballero J. Alonso R. Ibañez V. Terol J. Talon M. Dopazo J. (2015). A phylogenetic analysis of 34 chloroplast genomes elucidates the relationships between wild and domestic species within the genus Citrus. *Mol. Biol. Evol.* 32 2015–2035. 10.1093/molbev/msv082 25873589 PMC4833069

[B8] Chang C. C. Lin H. C. Lin I. P. Chow T. Y. Chen H. H. Chen W. H. (2006). The chloroplast genome of *Phalaenopsis aphrodite* (Orchidaceae): comparative analysis of evolutionary rate with that of grasses and its phylogenetic implications. *Mol. Biol. Evol.* 23 279–291. 10.1093/molbev/msj029 16207935

[B9] Cho K. S. Yun B. K. Yoon Y. H. Hong S. Y. Mekapogu M. Kim K. H. (2015). Complete chloroplast genome sequence of tartary buckwheat (*Fagopyrum tataricum*) and comparative analysis with common buckwheat (*F. esculentum)*. *PLoS One* 10:e0125332. 10.1371/journal.pone.0125332 25966355 PMC4428892

[B10] Coissac E. Hollingsworth P. M. Lavergne S. Taberlet P. (2016). From barcodes to genomes: extending the concept of DNA barcoding. *Mol. Ecol.* 25 1423–1428. 10.1111/mec.13549 26821259

[B11] Drummond A. J. Ho S. Y. Phillips M. J. Rambaut A. (2006). Relaxed phylogenetics and dating with confidence. *PLoS Biol.* 4:e88. 10.1371/journal.pbio.0040088 16683862 PMC1395354

[B12] Drummond A. J. Suchard M. A. Xie D. Rambaut A. (2012). Bayesian phylogenetics with BEAUti and the BEAST 1.7. *Mol. Biol. Evol.* 29 1969–1973. 10.1093/molbev/mss075 22367748 PMC3408070

[B13] Favre A. Michalak I. Chen C. H. Wang J. C. Pringle J. S. Matuszak H. (2016). Out-of-Tibet: the spatio-temporal evolution of *Gentiana* (Gentianaceae). *J. Biogeogr.* 43 1967–1978. 10.1111/jbi.12840

[B14] Favre A. Yuan Y. M. Küpfer P. Alvarez N. (2010). Phylogeny of subtribe Gentianinae (Gentianaceae): biogeographic inferences despite limitations in temporal calibration points. *Taxon* 59 1701–1711. 10.2307/41059867

[B15] Frazer K. A. Pachter L. Poliakov A. Rubin E. M. Dubchak I. (2004). VISTA: computational tools for comparative genomics. *Nucleic Acids Res.* 32 W273–W279. 10.1093/nar/gkh458 15215394 PMC441596

[B16] Fu P. C. Zhang Y. Z. Geng H. M. Chen S. L. (2016). The complete chloroplast genome sequence of *Gentiana lawrencei* var. farreri (Gentianaceae) and comparative analysis with its congeneric species. *PeerJ* 4:e2540. 10.7717/peerj.2540 27703869 PMC5047142

[B17] Guindon S. Gascuel O. (2003). A simple, fast and accurate method to estimate large phylogenies by maximum-likelihood. *Syst. Biol.* 52 696–704. 10.1080/10635150390235520 14530136

[B18] He T. N. (1988). “Sect. Cruciata,” in *Flora Reipublicae Popularis Sinicae 62. Gentianaceae* ed. He T. N. (Beijing: Science Press) 1–75.

[B19] Ho T. N. (1985). A study on the genus Gentiana of China (IV). *Bull. Bot. Res.* 5 1–22.

[B20] Ho T. N. Liu S. W. (2001). *A Worldwide Monograph of Gentiana.* Beijing: Science Press.

[B21] Ho T. N. Liu S. W. Lu X. F. (1996). A phylogenetic analysis of *Gentiana* (Gentianaceae). *Acta Phytotaxonomica Sin.* 34 505–506.

[B22] Ho T. N. Pringle J. S. (1995). “Gentianaceae,” in *Flora of China* Vol. 16 eds Wu Z. Y. Raven P. H. (Beijing: Science Press), 1–140.

[B23] Jansen R. K. Raubeson L. A. Boore J. L. dePamphilis C. W. Chumley T. W. Haberle R. C. (2005). Methods for obtaining and analyzing chloroplast genome sequences. *Methods Enzymol* 395 348–384. 10.1016/S0076-6879(05)95020-9 15865976

[B24] Kearse M. Moir R. Wilson A. Stones-Havas S. Cheung M. Sturrock S. (2012). Geneious Basic: an integrated and extendable desktop software platform for the organization and analysis of sequence data. *Bioinformatics* 28 1647–1649. 10.1093/bioinformatics/bts199 22543367 PMC3371832

[B25] Khan G. Zhang F. Q. Gao Q. B. Fu P. C. Xing R. Wang J. L. (2016). Phylogenetic analyses of *Spiraea*, (Rosaceae) distributed in the Qinghai-Tibetan Plateau and adjacent regions: insights from molecular data. *Plant Syst. Evol.* 302 11–21. 10.1007/s00606-015-1238-6

[B26] Kim H. T. Kim J. S. Moore M. J. Neubig K. M. Williams N. H. Whitten W. M. (2015). Seven new complete plastome sequences reveal rampant independent loss of the ndh gene family across orchids and associated instability of the inverted repeat/small single-copy region boundaries. *PLoS One* 10:e0142215. 10.1371/journal.pone.0142215 26558895 PMC4641739

[B27] Li X. Yang Y. Henry R. J. Rossetto M. Wang Y. Chen S. (2015). Plant DNA barcoding: from gene to genome. *Biol. Rev.* 90 157–166. 10.1111/brv.12104 24666563

[B28] Li X. J. Wang L. Y. Yang H. L. Liu J. Q. (2007). Confirmation of natural hybrids between *Gentiana straminea* and *G. siphonantha* (Gentianaceae) based on molecular evidence. *Front. Biol. China* 3:470–476. 10.1007/s11515-008-0076-0

[B29] Librado P. Rozas J. (2009). DnaSP v5: a software for comprehensive analysis of DNA polymorphism data. *Bioinformatics* 25 1451–1452. 10.1093/bioinformatics/btp187 19346325

[B30] Lin C. S. Chen J. J. Huang Y. T. Chan M. T. Daniell H. Chang W. J. (2015). The location and translocation of ndh genes of chloroplast origin in the Orchidaceae family. *Sci. Rep.* 5:9040. 10.1038/srep09040 25761566 PMC4356964

[B31] Liu J. Q. Duan Y. W. Hao G. Ge X. J. Sun H. (2014). Evolutionary history and underlying adaptation of alpine plants on the Qinghai-Tibet Plateau. *J. Syst. Evol.* 52 241–249. 10.1111/jse.12094

[B32] Lohse M. Drechsel O. Bock R. (2007). OrganellarGenomeDRAW (OGDRAW): a tool for the easy generation of high-quality custom graphical maps of plastid and mitochondrial genomes. *Curr. Genet.* 52 267–274. 10.1007/s00294-007-0161-y 17957369

[B33] Martín M. Sabater B. (2010). Plastid ndh genes in plant evolution. *Plant Physiol. Biochem.* 48 636–645. 10.1016/j.plaphy.2010.04.009 20493721

[B34] Martin W. Stoebe B. Goremykin V. Hansmann S. Hasegawa M. Kowallik K. V. (1998). Gene transfer to the nucleus and the evolution of chloroplasts. *Nature* 393 162–165. 10.1038/30234 11560168

[B35] Millen R. S. Olmstead R. G. Adams K. L. Palmer J. D. Lao N. T. Heggie L. (2001). Many parallel losses of infA from chloroplast DNA during angiosperm evolution with multiple independent transfers to the nucleus. *Plant Cell* 13 645–658. 10.1105/tpc.13.3.645 11251102 PMC135507

[B36] Mishiba K. I. Yamane K. Nakatsuka T. Nakano Y. Yamamura S. Abe J. (2009). Genetic relationships in the genus *Gentiana* based on chloroplast DNA sequence data and nuclear DNA content. *Breed. Sci.* 59 119–127. 10.1270/jsbbs.59.119

[B37] Ni L. Zhao Z. Xu H. Chen S. Dorje G. (2016a). Chloroplast genome structures in *Gentiana* (Gentianaceae), based on three medicinal alpine plants used in Tibetan herbal medicine. *Curr. Genet.* 63 241–252. 10.1007/s00294-016-0631-1 27422574

[B38] Ni L. Zhao Z. Xu H. Chen S. Dorje G. (2016b). The complete chloroplast genome of *Gentiana straminea* (Gentianaceae), an endemic species to the Sino-Himalayan subregion. *Gene* 577 281–288. 10.1016/j.gene.2015.12.005 26680100

[B39] Nikiforova S. V. Cavalieri D. Velasco R. Goremykin V. (2013). Phylogenetic analysis of 47 chloroplast genomes clarifies the contribution of wild species to the domesticated apple maternal line. *Mol. Biol. Evol.* 30 1751–1760. 10.1093/molbev/mst092 23676769

[B40] Niu Z. Xue Q. Zhu S. Sun J. Liu W. Ding X. (2017). The complete plastome sequences of four Orchid species: insights into evolution of the Orchidaceae and the utility of plastomic mutational hotspots. *Front. Plant Sci.* 8:715. 10.3389/fpls.2017.00715 28515737 PMC5413554

[B41] Palmer J. D. (1985). Comparative organization of chloroplast genomes. *Annu. Rev. Genet.* 19 325–354. 10.1146/annurev.ge.19.120185.001545 3936406

[B42] Peredo E. L. King U. M. Les D. H. (2013). The plastid genome of Najas flexilis: adaptation to submersed environments is accompanied by the complete loss of the NDH complex in an aquatic angiosperm. *PLoS One* 8:e68591. 10.1371/journal.pone.0068591 23861923 PMC3701688

[B43] Pirie M. D. Litsios G. Bellstedt D. U. Salamin N. Kissling J. (2015). Back to Gondwanaland: can ancient vicariance explain (some) Indian Ocean disjunct plant distributions? *Biol. Lett.* 11:20150086. 10.1098/rsbl.2015.0086 26063747 PMC4528461

[B44] Posada D. (2008). jModelTest: phylogenetic model averaging. *Mol. Biol. Evol.* 25 1253–1256. 10.1093/molbev/msn083 18397919

[B45] Rambaut A. Drummond A. J. (2010). *Tracer, Version 1.5.* Available at: http://tree.bio.ed.ac.uk/software/tracer/ [accessed August 25, 2013].

[B46] Ross T. G. Barrett C. F. Soto Gomez M. Lam V. K. Henriquez C. L. Les D. H. (2015). Plastid phylogenomics and molecular evolution of Alismatales. *Cladistics* 32 160–178. 10.1111/cla.12133 34736309

[B47] Ruhlman T. A. Chang W. J. Chen J. J. Huang Y. T. Chan M. T. Jin Z. (2015). NDH expression marks major transitions in plant evolution and reveals coordinate intracellular gene loss. *BMC Plant Biol* 15:100. 10.1186/s12870-015-0484-7 25886915 PMC4404220

[B48] Rybczyński J. J. Davey M. R. Mikuła A. (eds) (2015). *The Gentianaceae-Volume 2: Biotechnology and Applications.* Berlin: Springer.

[B49] Sanderson M. J. Copetti D. Búrquez A. Bustamante E. Charboneau J. L. Eguiarte L. E. (2015). Exceptional reduction of the plastid genome of saguaro cactus (*Carnegiea gigantea*): loss of the ndh gene suite and inverted repeat. *Am. J. Bot.* 102 1115–1127. 10.3732/ajb.1500184 26199368

[B50] Sazanov L. A. Burrows P. A. Nixon P. J. (1998). The plastid ndh genes code for an NADH-specific dehydrogenase: isolation of a complex I analogue from pea thylakoid membranes. *Proc. Natl. Acad. Sci. U.S.A.* 95 1319–1324. 10.1073/pnas.95.3.1319 9448329 PMC18756

[B51] Shaw J. Shafer H. L. Leonard O. R. Kovach M. J. Schorr M. Morris A. B. (2014). Chloroplast DNA sequence utility for the lowest phylogenetic and phylogeographic inferences in angiosperms: the tortoise and the hare IV. *Am. J. Bot.* 101 1987–2004. 10.3732/ajb.1400398 25366863

[B52] Shepherd L. D. Lange P. J. D. Perrie L. R. Heenan P. B. (2017). Chloroplast phylogeography of new zealand sophora trees (Fabaceae): extensive hybridization and widespread last glacial maximum survival. *J. Biogeogr.* 44 1640–1651. 10.1111/jbi.12963

[B53] Shinozaki K. Ohme M. Tanaka M. Wakasugi T. Hayashida N. Matsubayashi T. (1986). The complete nucleotide sequence of the tobacco chloroplast genome: its gene organization and expression. *EMBO J.* 5 2043–2049. 10.1007/BF02669253 16453699 PMC1167080

[B54] Stefanović S. Olmstead R. G. (2005). Down the slippery slope: plastid genome evolution in convolvulaceae. *J. Mol. Evol.* 61 292–305. 10.1007/s00239-004-0267-5 15999247

[B55] Takayama K. Tamura M. Tateishi Y. Webb E. L. Kajita T. (2013). Strong genetic structure over the American continents and transoceanic dispersal in the mangrove genus *Rhizophora* (Rhizophoraceae) revealed by broad-scale nuclear and chloroplast DNA analysis. *Am. J. Bot.* 100 1191–1201. 10.3732/ajb.1200567 23711904

[B56] Wakasugi T. Tsudzuki J. Ito S. Nakashima K. Tsudzuki T. Sugiura M. (1994). Loss of all ndh genes as determined by sequencing the entire chloroplast genome of the black pine Pinus thunbergii. *Proc. Natl. Acad. Sci. U.S.A.* 91 9794–9798. 10.1073/pnas.91.21.9794 7937893 PMC44903

[B57] Wang P. Duan W. Takabayashi A. Endo T. Shikanai T. Ye J. Y. (2006). Chloroplastic NAD (P)H dehydrogenase in tobacco leaves functions in alleviation of oxidative damage caused by temperature stress. *Plant Physiol.* 141 465–474. 10.1104/pp.105.070490 16428601 PMC1475475

[B58] Wen J. Zhang J.-Q. Nie Z.-L. Zhong Y. Sun H. (2014). Evolutionary diversifications of plants on the Qinghai-Tibetan Plateau. *Front. Genet.* 5:4 10.3389/fgene.2014.00004 PMC3921583 24575120

[B59] Wolfe K. H. Li W. H. Sharp P. M. (1987). Rates of nucleotide substitution vary greatly among plant mitochondrial, chloroplast, and nuclear DNAs. *Proc. Natl. Acad. Sci. U.S.A.* 84 9054–9058. 10.1073/pnas.84.24.9054 3480529 PMC299690

[B60] Wu C. S. Wang Y. N. Hsu C. Y. Lin C. P. Chaw S. M. (2011). Loss of different inverted repeat copies from the chloroplast genomes of Pinaceae and Cupressophytes and influence of heterotachy on the evaluation of gymnosperm phylogeny. *Genome Biol. Evol.* 3 1284–1295. 10.1093/gbe/evr095 21933779 PMC3219958

[B61] Wyman S. K. Janse R. K. Boore J. L. (2004). Automatic annotation of organellar genomes with DOGMA. *Bioinformatics* 20 3252–3255. 10.1093/bioinformatics/bth352 15180927

[B62] Yang J. B. Tang M. Li H. T. Zhang Z. R. Li D. Z. (2013). Complete chloroplast genome of the genus Cymbidium: lights into the species identification, phylogenetic implications and population genetic analyses. *BMC Evol. Biol.* 13:84. 10.1186/1471-2148-13-84 23597078 PMC3644226

[B63] Zerbino D. R. Birney E. (2008). Velvet: algorithms for de novo short read assembly using de Bruijn graphs. *Genome Res.* 18 821–829. 10.1101/gr.074492.107 18349386 PMC2336801

[B64] Zhang M. Y. Fritsch P. W. Ma P. F. Wang H. Lu L. Li D. Z. (2017). Plastid phylogenomics and adaptive evolution of *Gaultheria* series *Trichophyllae* (Ericaceae), a clade from sky islands of the Himalaya-Hengduan Mountains. *Mol. Phylogenet. Evol.* 110 7–18. 10.1016/j.ympev.2017.01.015 28215572

[B65] Zhang S. D. Jin J. J. Chen S. Y. Chase M. W. Soltis D. E. Li H. T. (2017). Diversification of Rosaceae since the Late Cretaceous based on plastid phylogenomics. *New Phytol.* 214 1355–1367. 10.1111/nph.14461 28186635

