# Supplementary material for: The Complete Plastome Sequences of Seven Species in Gentiana sect. Kudoa (Gentianaceae): Insights Into Plastid Gene Loss and Molecular Evolution
Source: Front Plant Sci. 2018 May 1;9:493. doi: 10.3389/fpls.2018.00493 (PMC5938401; doi:10.3389/fpls.2018.00493)
Supplement: Supplementary file 1 [file Image_1.PDF]

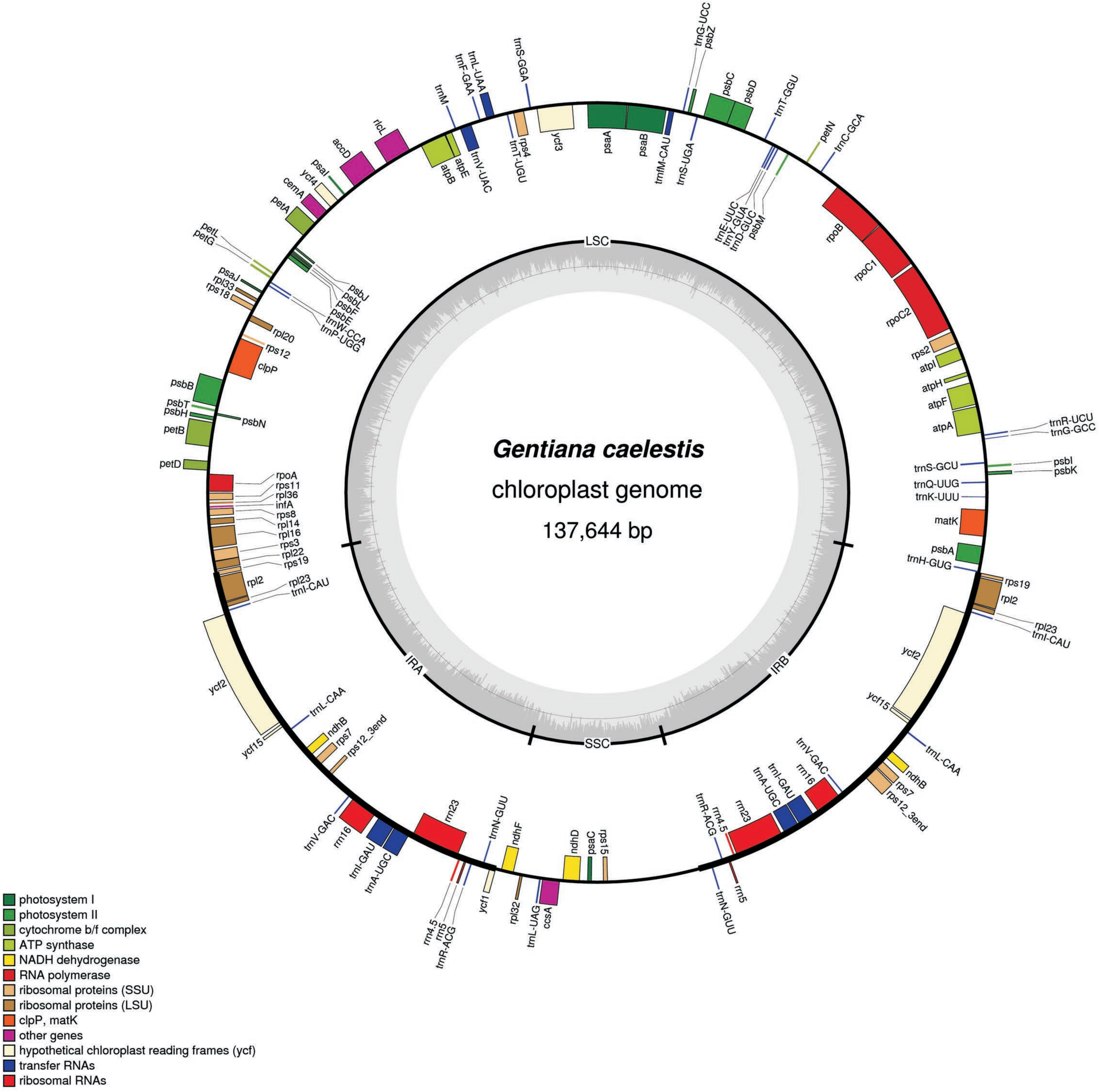

Gentiana caelestis
chloroplast genome
137,644 bp
LSC
IRA
IRB
SSC
trnG-UCC
psbZ
psbC
psbD
trnT-GGU
petN
trnC-GCA
trnS-GGA
trnL-UAA
trnF-GAA
trnM
rbcL
accD
psaI
ycf4
cemA
petA
atpB
atpE
trnV-UAC
trnT-UGU
rps4
ycf3
psaA
psaB
trnfM-CAU
trnS-UGA
trnE-UUC
trnY-GUA
trnD-GUC
psbM
rpoB
rpoC1
rpoC2
rps2
atpI
atpH
atpF
atpA
trnR-UCU
trnG-GCC
trnS-GCU
psbI
psbK
trnQ-UUG
trnK-UUU
matK
psbA
trnH-GUG
rps19
rpl2
rpl23
trnI-CAU
ycf2
ycf15
trnL-CAA
ndhB
rps7
rps12_3end
trnV-GAC
rrn16
trnI-GAU
trnA-UGC
rrn23
rrn4.5
rrn5
trnR-ACG
trnN-GUU
ycf1
ndhF
rpl32
trnL-UAG
ccsA
ndhD
psaC
rps15
petL
petG
psaJ
rpl33
rps18
psbJ
psbL
psbF
psbE
trnW-CCA
trnP-UGG
rpl20
rps12
clpP
psbB
psbT
psbH
psbN
petB
petD
rpoA
rps11
rpl36
infA
rps8
rpl14
rpl16
rps3
rpl22
photosystem I
photosystem II
cytochrome b/f complex
ATP synthase
NADH dehydrogenase
RNA polymerase
ribosomal proteins (SSU)
ribosomal proteins (LSU)
clpP, matK
other genes
hypothetical chloroplast reading frames (ycf)
transfer RNAs
ribosomal RNAs
