# Supplementary material for: The Complete Plastome Sequences of Seven Species in Gentiana sect. Kudoa (Gentianaceae): Insights Into Plastid Gene Loss and Molecular Evolution
Source: Front Plant Sci. 2018 May 1;9:493. doi: 10.3389/fpls.2018.00493 (PMC5938401; doi:10.3389/fpls.2018.00493)
Supplement: Supplementary file 3 [file Image_3.PDF]

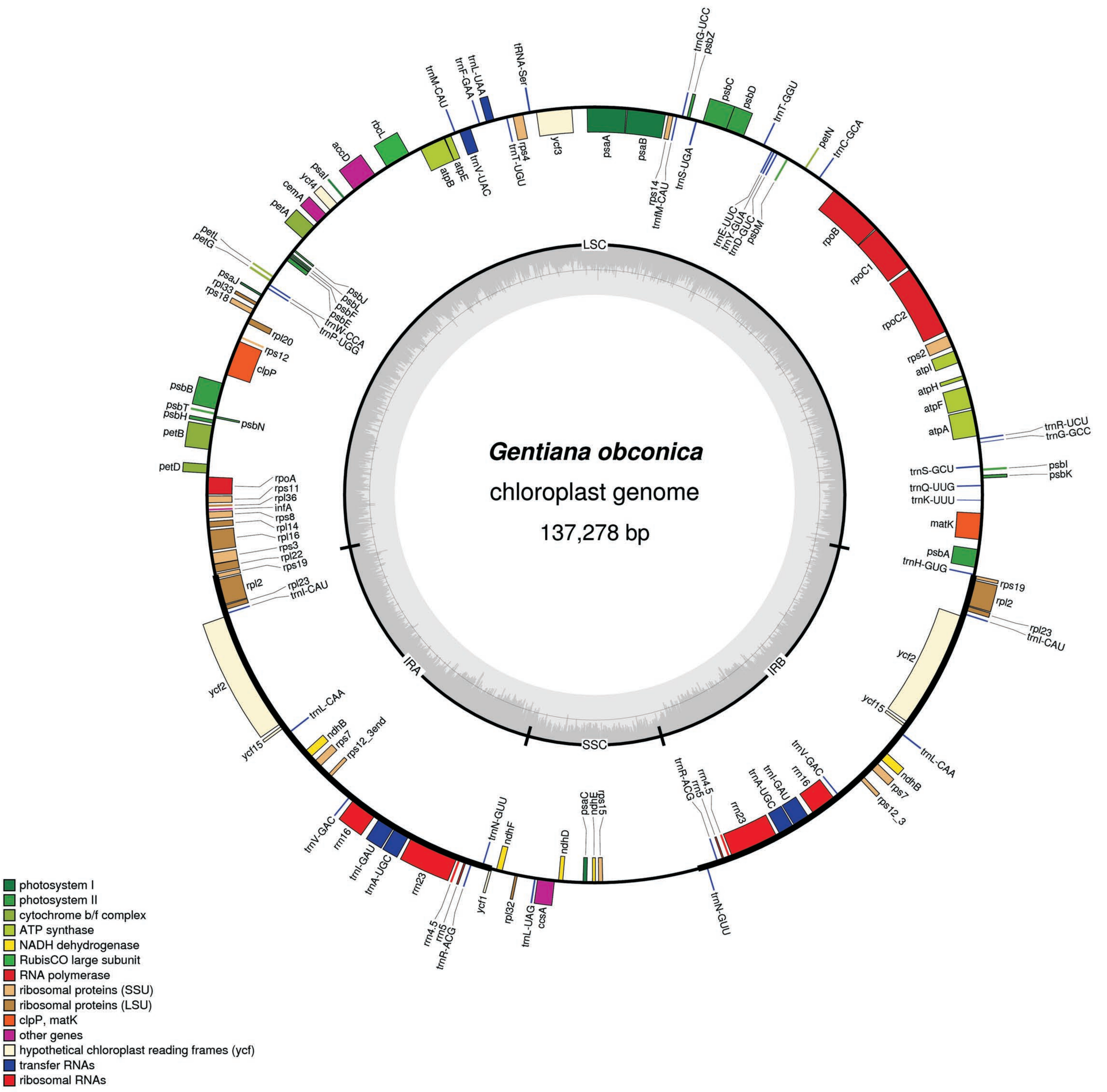
Gentiana obconica
chloroplast genome
137,278 bp
LSC
IRA
IRB
SSC
trnG-UCC
psbZ
psbC
psbD
trnT-GGU
petN
trnC-GCA
rpoB
rpoC1
rpoC2
rps2
atpI
atpH
atpF
atpA
trnR-UCU
trnG-GCC
trnS-GCU
psbI
psbK
trnQ-UUG
trnK-UUU
matK
psbA
trnH-GUG
rps19
rpl2
rpl23
trnI-CAU
ycf2
ycf15
trnL-CAA
ndhB
rps7
rps12_3
trnV-GAC
rrn16
trnI-GAU
trnA-UGC
rrn23
rrn4.5
rrn5
trnR-ACG
trnN-GUU
psaC
ndhE
rps15
ndhD
ccsA
trnL-UAG
rpl32
ndhF
ycf1
rps12_3end
rpoA
rps11
rpl36
infA
rps8
rpl14
rpl16
rps3
rpl22
petD
petB
psbH
psbT
psbN
psbB
clpP
rps12
rpl20
rps18
rpl33
psaJ
petG
petL
psbJ
psbL
psbF
psbE
trnW-CCA
trnP-UGG
petA
cemA
ycf4
psaI
accD
rbcL
atpB
atpE
trnM-CAU
trnF-GAA
trnL-UAA
trnV-UAC
trnT-UGU
rps4
tRNA-Ser
ycf3
psaA
psaB
rps14
trnfM-CAU
trnS-UGA
trnE-UUC
trnY-GUA
trnD-GUC
psbM
photosystem I
photosystem II
cytochrome b/f complex
ATP synthase
NADH dehydrogenase
RubisCO large subunit
RNA polymerase
ribosomal proteins (SSU)
ribosomal proteins (LSU)
clpP, matK
other genes
hypothetical chloroplast reading frames (ycf)
transfer RNAs
ribosomal RNAs
