# Supplementary figures and images for: The Complete Plastome Sequences of Seven Species in Gentiana sect. Kudoa (Gentianaceae): Insights Into Plastid Gene Loss and Molecular Evolution
Source: Front Plant Sci. 2018 May 1;9:493. doi: 10.3389/fpls.2018.00493 (PMC5938401; doi:10.3389/fpls.2018.00493)

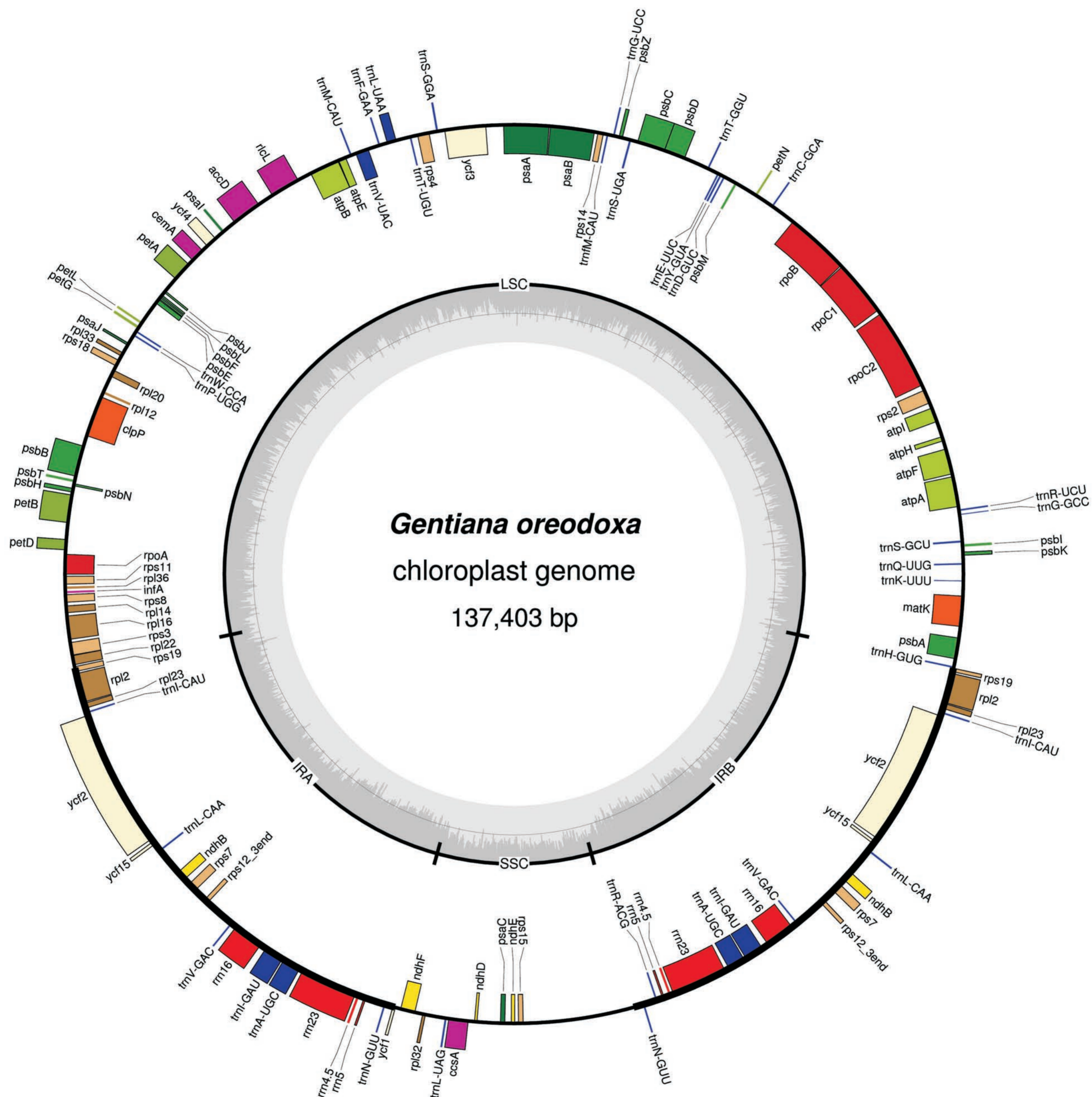

Supplement: Supplementary file 4 [file Image_4.PDF]

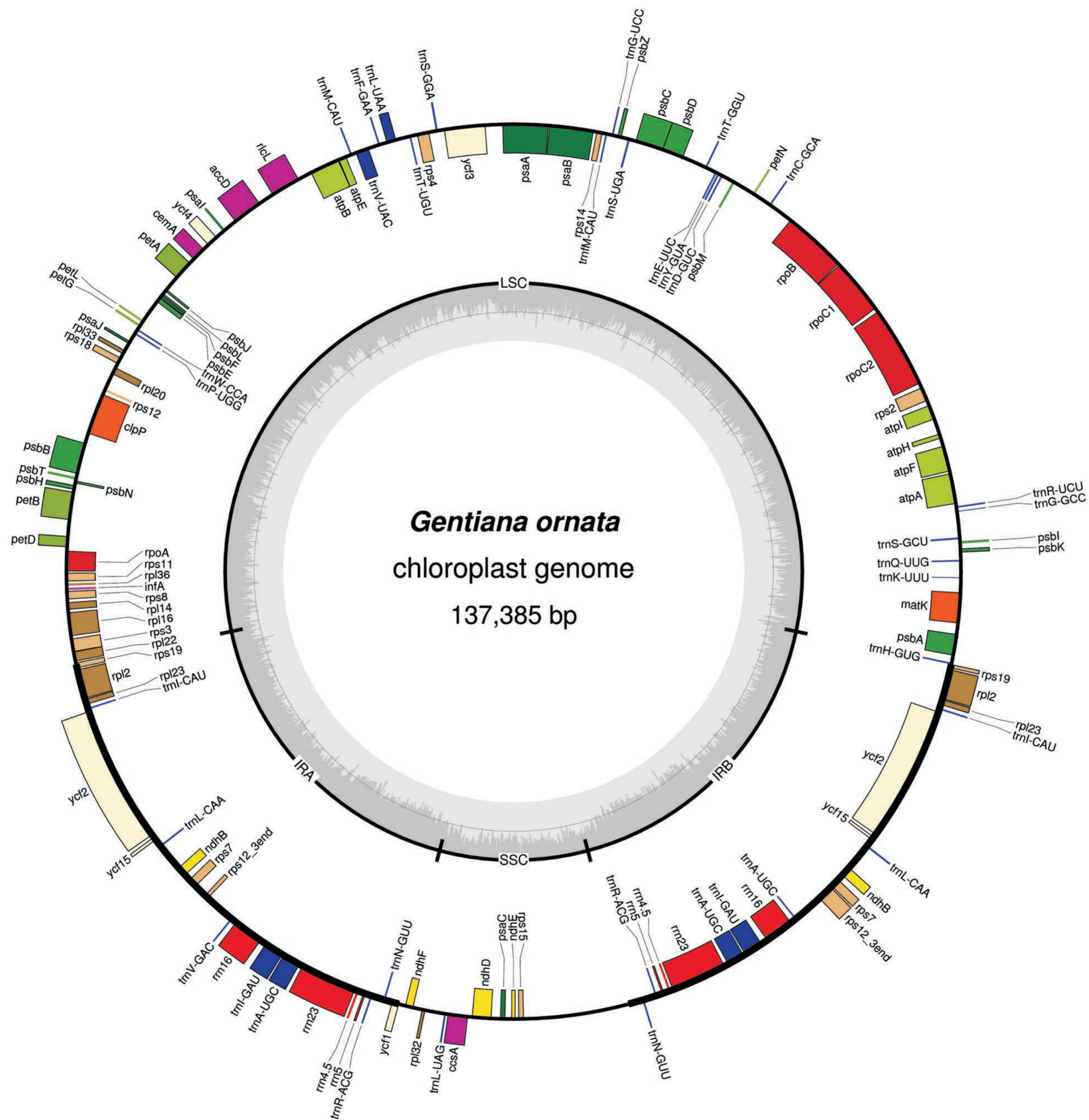

Supplement: Supplementary file 5 [file Image_5.PDF]

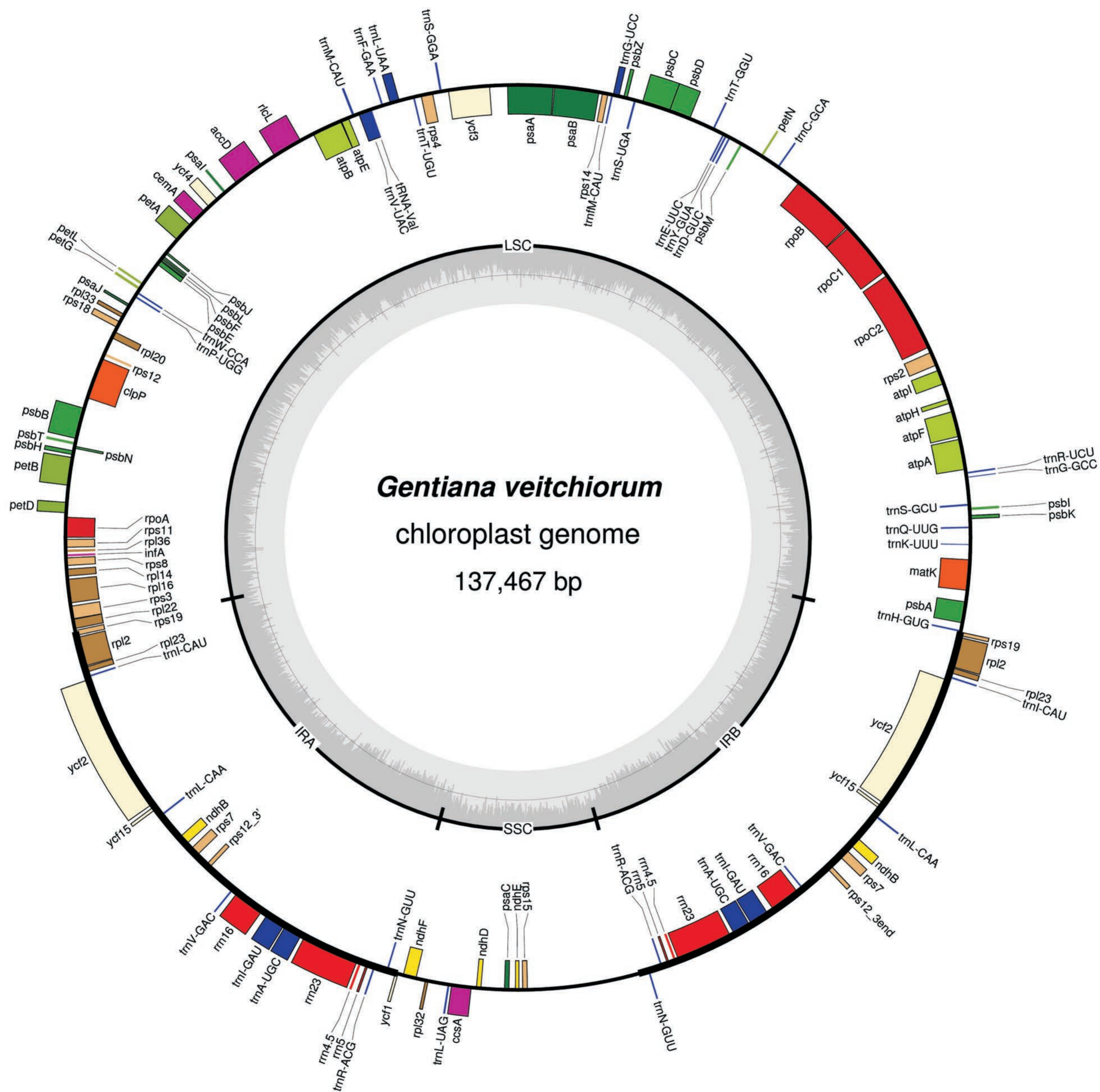

Supplement: Supplementary file 7 [file Image_7.PDF]
