# Supplementary material for: The Complete Plastome Sequences of Seven Species in Gentiana sect. Kudoa (Gentianaceae): Insights Into Plastid Gene Loss and Molecular Evolution
Source: Front Plant Sci. 2018 May 1;9:493. doi: 10.3389/fpls.2018.00493 (PMC5938401; doi:10.3389/fpls.2018.00493)
Supplement: Supplementary file 6 [file Image_6.PDF]

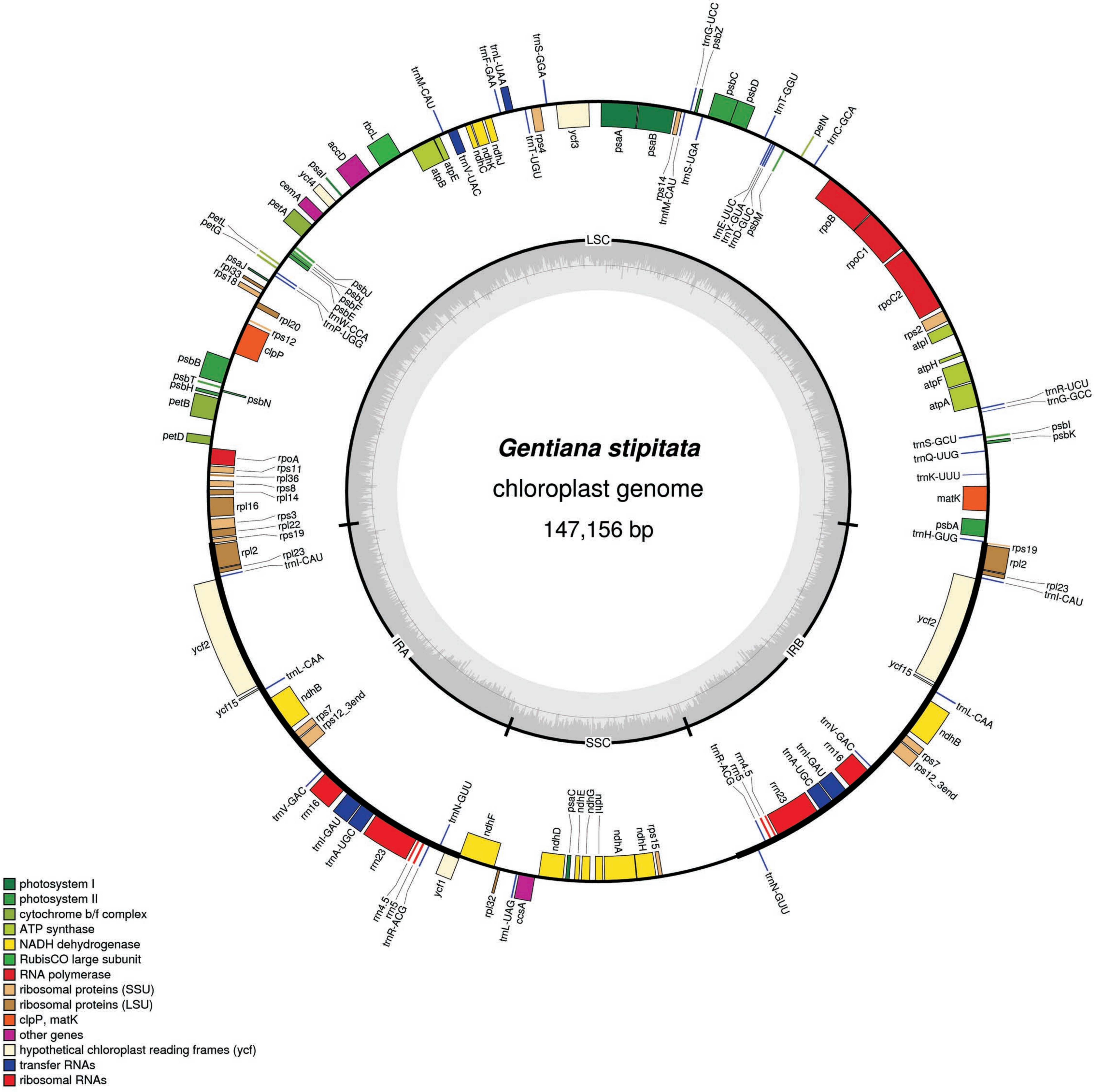

Gentiana stipitata
chloroplast genome
147,156 bp
LSC
IRA
IRB
SSC
trnM-CAU
trnL-UAA
trnF-GAA
trnS-GGA
trnG-UCC
psbZ
psbC
psbD
trnT-GGU
petN
trnC-GCA
rpoB
rpoC1
rpoC2
rps2
atpI
atpH
atpF
atpA
trnR-UCU
trnG-GCC
psbI
psbK
trnS-GCU
trnQ-UUG
trnK-UUU
matK
psbA
trnH-GUG
rps19
rpl2
rpl23
trnI-CAU
ycf2
ycf15
trnL-CAA
ndhB
rps7
rps12_3end
trnV-GAC
rrn16
trnI-GAU
trnA-UGC
rrn23
rrn4.5
rrn5
trnR-ACG
trnN-GUU
rps15
ndhH
ndhA
ndhI
ndhG
ndhE
psaC
ndhD
ccsA
trnL-UAG
rpl32
ndhF
ycf1
trnN-GUU
rrn4.5
rrn5
trnR-ACG
rrn23
trnA-UGC
trnI-GAU
rrn16
trnV-GAC
ycf15
ycf2
trnL-CAA
ndhB
rps7
rps12_3end
rpl23
trnI-CAU
rpl2
rps19
rpl22
rps3
rpl16
rpl14
rps8
rpl36
rps11
rpoA
petD
petB
psbH
psbT
psbN
psbB
clpP
rps12
rpl20
rps18
rpl33
psaJ
trnP-UGG
trnW-CCA
psbE
psbF
psbL
psbJ
petG
petL
petA
cemA
ycf4
psaI
accD
rbcL
atpB
atpE
trnV-UAC
ndhC
ndhK
ndhJ
trnT-UGU
rps4
ycf3
psaA
psaB
rps14
trnfM-CAU
trnS-UGA
trnE-UUC
trnY-GUA
trnD-GUC
psbM
photosystem I
photosystem II
cytochrome b/f complex
ATP synthase
NADH dehydrogenase
RubisCO large subunit
RNA polymerase
ribosomal proteins (SSU)
ribosomal proteins (LSU)
clpP, matK
other genes
hypothetical chloroplast reading frames (ycf)
transfer RNAs
ribosomal RNAs
