# Supplementary material for: The Complete Plastome Sequences of Seven Species in Gentiana sect. Kudoa (Gentianaceae): Insights Into Plastid Gene Loss and Molecular Evolution
Source: Front Plant Sci. 2018 May 1;9:493. doi: 10.3389/fpls.2018.00493 (PMC5938401; doi:10.3389/fpls.2018.00493)
Supplement: Supplementary file 8 [file Table_1.DOCX]

Table S1 The information of samples used in this study.

| Taxa | Voucher ref. | Locality | Latitude (N) | Longitude (E) | Altitude (m/a.s.l) |
| --- | --- | --- | --- | --- | --- |
| *G.caelestis* | Fu2016092 | Kangding, Sichuan | 29°53'51'' | 102°00'43'' | 4002 |
| *G.obconica* | Fu2016190 | Linzhi, Tibet | 29°37**'**09'' | 94°40'07'' | 4434 |
| *G.oreodoxa* | Fu2016188 | Basu, Tibet | 29°51'02'' | 96°41'52'' | 3950 |
| *G.ornata* | Fu2016164 | Daocheng, Sichuan | 29°27'09'' | 100°13'27'' | 4657 |
| *G.veitchiorum* | Fu2016191 | Linzhi, Tibet | 29°37'09'' | 94°40'07'' | 4434 |
| *G.hexaphylla* | Fu2016027 | Aba, Sichuan | 32°45'21'' | 102°06'55'' | 3937 |
| *G.stipitata* | Fu2016061 | Dege, Sichuan | 31°59'24'' | 99°05'13'' | 4021 |

Table S2 The information of the raw reads in this study.

| Sample | Clean reads | Clean bases | Q30(%) |
| --- | --- | --- | --- |
| *G.caelestis* | 76053836 | 11179563107 | 97.512 |
| *G.obconica* | 67713180 | 9929381451 | 97.571 |
| *G.oreodoxa* | 87359644 | 12748711811 | 96.885 |
| *G.ornata* | 49564862 | 7282389826 | 98.007 |
| *G.veitchiorum* | 80717702 | 11830553534 | 96.506 |
| *G.hexaphylla* | 85428300 | 12471186417 | 96.430 |
| *G.stipitata* | 77644996 | 11402610861 | 96.400 |

Table S3 List of all pairs of primers used for assembly validation.

| Taxa | Primer Name | Sequences (5'-3') |
| --- | --- | --- |
| *G.caelestis* | *GenCp6-2F* | ATTCCATTCATTTAATATCCCT |
|  | *GenCp6-2R* | CTAATAACTTGGTCCACTGCGG |
| *G.obconica* | *GenCp7-1F* | CTTCATAGGACCGTCCACGAAT |
|  | *GenCp7-1R* | GTAGAAAAAAACCCACAACCCC |
|  | *GenCp7-2F* | ATTCCTGTTCTCAACCCATGAC |
|  | *GenCp7-2R* | GCTAAATCTGCACAATTTCCCC |
| *G.oreodoxa* | *GenCp8-1F* | ATCCTCTTGTAAATGCTCTAAA |
|  | *GenCp8-1R* | GAAATAATCGTAACCTGGTCCC |
|  | *GenCp8-2F* | ATTCCATTCATTTAATATCCCT |
|  | *GenCp8-2R* | CATTTCTGCTCTTATACACGCT |
| *G.ornata* | *GenCp5-2F* | TTATGGATTTCTGACCACATTC |
|  | *GenCp5-2R* | TTTCTGCTCTTATACACGCTGC |
|  | *GenCp5-4F* | ACGGGGGGGGTGAGGGGAGAGC |
|  | *GenCp5-4R* | ACGGATAGTCAAGGGGCGGATG |
| *G.veitchiorum* | *GenCp4-1F* | TGAGATTCATGGCAATTCGGAT |
|  | *GenCp4-1F* | TAGCGCGTTTGTTTTGGGTACA |
|  | *GenCp4-3F* | ATTCCTGTTCTCAACCCATGAC |
|  | *GenCp4-3R* | GCTAAATCTGCACAATTTCCCC |
|  | *GenCp4-4F* | CTTTGTGCTTGTTTAGTCCCCT |
|  | *GenCp4-4R* | GATAATCAATTCGGTCGTTGTG |
| *G.hexaphylla* | *GenCp1-2F1* | AAATAAGGGAGGTTTGTGAT |
|  | *GenCp1-2R1* | TTTAGCCTGCTTTTGGTCTA |
|  | *GenCp1-2F2* | TCCTGTTCTCAACCCATGAC |
|  | *GenCp1-2R2* | GCTAAATCTGCACAATTCCC |
|  | *GenCp1-4F* | TACATAACATAGAAATCACACT |
|  | *GenCp1-4R* | CTAAGATATAAAGAAGAAAAAG |
| *G.stipitata* | *2Gstipi1-F* | TGGAAAGTGAGGAAGAAAGAGA |
|  | *2Gstipi1-R* | GAGGAGTAAGAAGATTAGGCGA |
|  | *2Gstipi2-F* | GAATGGTATGAGTTTGTGAAAG |
|  | *2Gstipi2-R* | GTGAGGAAGAAAGAGATCTAGG |
|  | *G2stip_F* | GAACATCTTTCACAATCCCTGG |
|  | *G2stip_R* | CCTATTTGGTCAAATACCTAGC |
| For all taxa | *trnL(UAA)* | GGTTCAAGTCCCTCTATCCCC |
|  | *trnF(GAA)* | GGTTCAAGTCCCTCTATCCCC |
|  | *trnS(GCU)* | GCCGCTTTAGTCCACTCAGC |
|  | *trnG(UCC)* | GAACGAATCACACTTTTACCAC |
|  | *rbcL-F* | ATGTCACCACAAACAGAAAC |
|  | *rbcL-R* | TCGCATGTACCTGCAGTAGC |
|  | *rpl20* | TTTGTTCTACGTCTCCGAGC |
|  | *rps12* | GTCGAGGAACATGTACTAGG |

Table S4 GeneBank accession numbers of 21 chloroplast genomes used for phylogenetic analysis.

| Taxon | Order | Family | GenBank ID |
| --- | --- | --- | --- |
| *Gentiana caelestis* | Gentianales | Gentianaceae | MG192304 |
| *Gentiana obconica* | Gentianales | Gentianaceae | MG192306 |
| *Gentiana oreodoxa* | Gentianales | Gentianaceae | MG192307 |
| *Gentiana ornata* | Gentianales | Gentianaceae | MG192308 |
| *Gentiana veitchiorum* | Gentianales | Gentianaceae | MG192310 |
| *Gentiana lawrencei* var. *farreri* | Gentianales | Gentianaceae | KX096882 |
| *Gentiana hexaphylla* | Gentianales | Gentianaceae | MG192305 |
| *Gentiana stipitata* | Gentianales | Gentianaceae | MG192309 |
| *Gentiana straminea* | Gentianales | Gentianaceae | KJ657732 |
| *Gentiana crassicaulis* | Gentianales | Gentianaceae | KJ676538 |
| *Gentiana robusta* | Gentianales | Gentianaceae | KT159969 |
| *Catharanthus roseus* | Gentianales | Apocynaceae | NC_021423 |
| *Rhazya stricta* | Gentianales | Apocynaceae | NC_024292 |
| *Nerium oleander* | Gentianales | Apocynaceae | NC_025656 |
| *Pentalinon luteum* | Gentianales | Apocynaceae | NC_025658 |
| *Oncinotis tenuiloba* | Gentianales | Apocynaceae | NC_025657 |
| *Cynanchum auriculatum* | Gentianales | Apocynaceae | NC_029460 |
| *Asclepias syriaca* | Gentianales | Apocynaceae | NC_022432 |
| *Coffea arabica* | Gentianales | Rubiaceae | NC_008535 |
| *Morinda officinalis* | Gentianales | Rubiaceae | NC_028009 |
| *Lactuca sativa* | Asterales | Asteraceae | NC_007578 |

Figure S1 The maps of the chloroplast genome of *Gentiana* *caelestis*. Genes drawn inside the circle are transcribed clockwise, and those outside are transcribed counterclockwise. Genes belonging to different functional groups are shown in different colors.

Figure S2 The maps of the chloroplast genome of *Gentiana* *hexaphylla*. Genes drawn inside the circle are transcribed clockwise, and those outside are transcribed counterclockwise. Genes belonging to different functional groups are shown in different colors.

Figure S3 The maps of the chloroplast genome of *Gentiana* *obconica*. Genes drawn inside the circle are transcribed clockwise, and those outside are transcribed counterclockwise. Genes belonging to different functional groups are shown in different colors.

Figure S4 The maps of the chloroplast genome of *Gentiana* *oreodoxa*. Genes drawn inside the circle are transcribed clockwise, and those outside are transcribed counterclockwise. Genes belonging to different functional groups are shown in different colors.

Figure S5 The maps of the chloroplast genome of *Gentiana* *ornata*. Genes drawn inside the circle are transcribed clockwise, and those outside are transcribed counterclockwise. Genes belonging to different functional groups are shown in different colors.

Figure S6 The maps of the chloroplast genome of *Gentiana* *stipitata*. Genes drawn inside the circle are transcribed clockwise, and those outside are transcribed counterclockwise. Genes belonging to different functional groups are shown in different colors.

Figure S7 The maps of the chloroplast genome of *Gentiana* *veitchiorum*. Genes drawn inside the circle are transcribed clockwise, and those outside are transcribed counterclockwise. Genes belonging to different functional groups are shown in different colors.
